# Feasibility of a brief intervention for medication-overuse headache in primary care – a pilot study

**DOI:** 10.1186/1756-0500-7-165

**Published:** 2014-03-20

**Authors:** Espen Saxhaug Kristoffersen, Jørund Straand, Michael Bjørn Russell, Christofer Lundqvist

**Affiliations:** 1 Department of General Practice, Institute of Health and Society, University of Oslo, Blindern, PO Box 1130, 0318 Oslo, Norway; 2 Health Service Research Centre, Research Centre, Akershus University Hospital, PO Box 95, 1478 Lørenskog, Norway; 3 Head and Neck Research Group, Research Centre, Akershus University Hospital, PO Box 95, 1478 Lørenskog, Norway; 4 Institute of Clinical Medicine, Akershus University Hospital, University of Oslo, Nordbyhagen, Norway; 5 Department of Neurology, Akershus University Hospital, Nordbyhagen, Norway

**Keywords:** Chronic headache, Medication-overuse headache, Migraine, Brief intervention, General practice, Severity of dependence scale, Feasibility study, Pilot study

## Abstract

**Background:**

Medication-overuse headache (MOH) is a common problem in primary care. Brief intervention (BI) has successfully been used for detoxification from overuse of alcohol and drugs. The aim of this pilot study was to develop and test methodology, acceptability and logistics for a BI for MOH in primary care.

**Findings:**

Observational feasibility study of an intervention in a Norwegian general practice population.

Six general practitioners (GPs) were recruited. A screening questionnaire for MOH was sent to all 18–50 year old patients on these GPs` list. GPs were taught BI, which was applied to MOH patients as follows: Severity of dependence scale (SDS) scores were collected and individual feedback was given of the relationship between the SDS, medication overuse and headache. Finally, advice to reduce medication was given. Patients were invited to a headache interview three months after the BI. Main outcomes were feedback from GPs/patients about the feasibility and logistics of the study design, screening/recruitment process, BI and headache interviews. Efficacy and patient-related outcomes were not focused. The patients reported a high degree of acceptability of the methodology. The GPs reported the BI to be feasible to implement within a busy practice and to represent a new and improved instrument for communication with MOH patients. The BI requires further testing in a randomised controlled trial (RCT) in order to provide evidence of efficacy.

**Conclusion:**

This feasibility study will be used to improve the BI for MOH and the design of a cluster-RCT.

**Trial registration:**

ClinicalTrials.gov: NCT01078012 (Initially registered as controlled efficacy trial but changed to observational study).

## Findings

### Background

Headache is a frequent symptom in primary care attendees, accounting for 4% of the consultations in the UK [1]. Headache prescription medications only partly account for the total medication use for headache since most patients buy over-the-counter (OTC) drugs [2-4]. Between two and five percent of the world’s population report chronic headache [5-7], and approximately 75% have been in contact with their GP [2,3]. Medication-overuse headache (MOH) is a major cause of chronic headache with prevalence in the general population of 1–1.5% [4,6,8-10], and is probably the most costly headache disorder [11]. By definition, MOH is chronic headache (≥15 days/month) with frequent medication use (Table 1) [12-15].

**Table 1:** **The International Classification of Headache Disorders, 2nd edition (ICHD - II) criteria for medication-overuse headache**[12-15]

**Medication-overuse headache (MOH)**	
A.	Headache present on ≥15 days/month
B.	Regular overuse for >3 months of one or more drugs that can be taken for acute and/or symptomatic treatment of headache
C.	Headache has developed or markedly worsened during medication overuse

For simple analgesics and for combination of acute medications the intake must be 15 days or more per month, for triptans, ergotamins, opioids and combination analgesics,10 days per month is enough to get the diagnosis of MOH.

There is no consensus on the management of MOH and an obvious need for evidence-based and cost-effective management strategies [10,16,17]. However, detoxification is generally accepted as the avenue to employ [18], since withdrawal of the overused medication(s) leads to an improvement of the headache, after an initial worsening for 1–2 weeks [17,19].

MOH has been suggested to include subgroups of both simple and complex cases, where some of the latter may show “dependency-like” behaviour [20-24].

Previous studies from our group have revealed that the Severity of Dependence Scale (SDS) [25] can detect MOH among subjects with chronic headache in a general population [26,27].

Brief Intervention (BI) is a well-known approach to identify and treat unhealthy alcohol use [28]. BI involves the use of an identification tool followed by feedback to the identified individual as being “at risk”. The final step is to give information suggesting cutting down the use of the particular substance to predefined “acceptable” levels [28]. In primary care settings, there is now substantial evidence of the benefits and cost-effectiveness of BI for alcohol and various other drugs [29-31].

We have previously reported data from an open, un-controlled study of MOH in the general population, which suggested that three out of four MOH subjects managed to reduce their medication intake after just short information [32]. Similar simple advice also works in specialist care settings [33,34].

We have developed a BI for management of MOH. Prior to the start-up of the pilot study we planned to include efficacy of the treatment versus controls as one outcome. However, due to cost and expected low power in a pilot study of reasonable size [35], we decided not to include a control arm but rather focus on testing the feasibility of the method observationally. It is strongly recommended to use information (process, resources, management and scientific) gathered in feasibility studies to modify the research methodology to avoid practical problems and potentially unexpected consequences of embarking directly on a full scale study [36-38].

The aim of this feasibility study was thus to develop and to test acceptability, practicability and logistics of using BI for MOH in primary care in order to prepare and improve a later full scale randomised controlled trial (RCT).

### Method

#### *Design*

**Figure 1:**
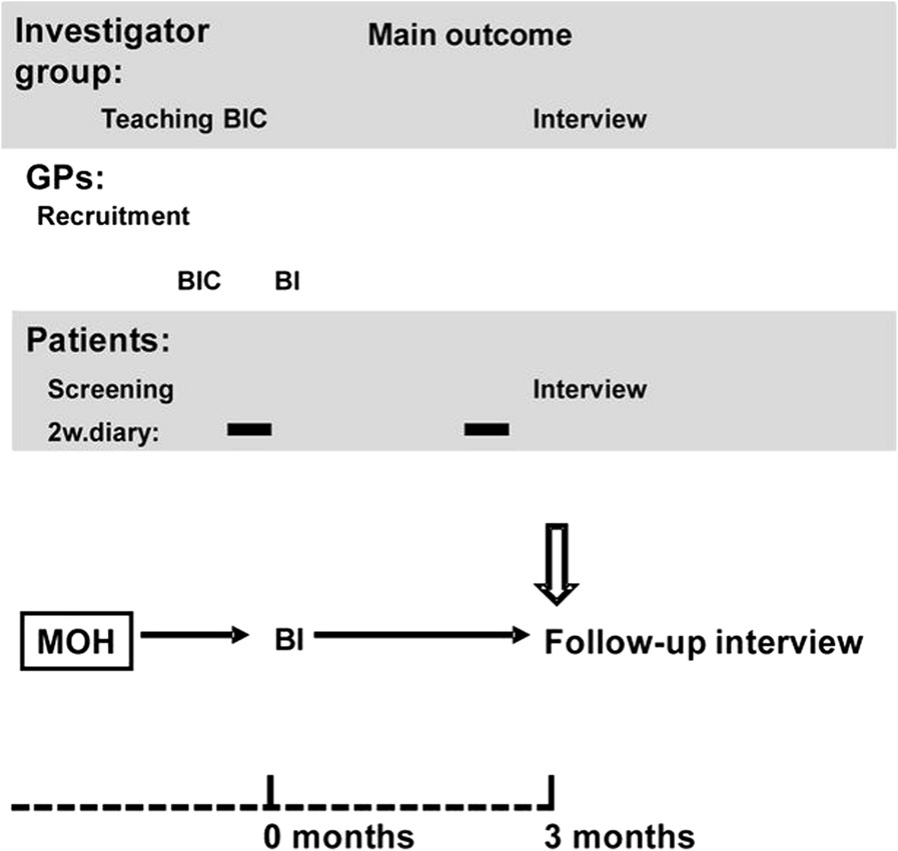
**Flowchart of study.** Figure illustrates main time line with the different phases with those mainly responsible for each phase (lower part). Upper part shows time-points for Patients data collection (2 week headache diaries (2w.diary) and interviews) as well as timing of various moments for the Investigator group and GPs with Brief Intervention training courses (BIC) for GPs and intervention. BI, Brief intervention; BIC, Brief Intervention course; GP, General practitioner.

Observational feasibility study of an intervention for MOH in a Norwegian general practice population (Figure 1).

##### 

**Participants (GPs and patients)** The study took place in Oslo in 2010.

The study population consisted of patients aged 18–50 on the patient lists of six GPs attending one continuous medical education (CME) group who had accepted to participate as a pilot group. Through the Norwegian GP list-patient system, all citizens are listed with a GP whether or not they have a reason to see their GP. A short validated headache screening questionnaire including questions about headache frequency, intensity (visual analogue scale (VAS), migraine and medication use was mailed to all 18–50 year old patients (N = 3575) on participating GP`s lists. Names and addresses were extracted from GP lists using specially designed software (Mediata Ltd, Tønsberg, Norway). All patients with self-reported headache ≥15 days/month and headache medication utilisation ≥10 days/month were invited to participate. Insufficient language skills and complex pain conditions were the only exclusion criteria. As this was a pilot study, no reminders were sent out. Screening-positive MOH patients invited into the intervention part of the study only received information that this was a study of headache care utilisation and that all participating patients would receive a headache interview and clinical examination by a headache expert. In addition, it was informed that some patients might be invited to a consultation by their GP. Patients were, thus, not informed that the focus was chronic headache and medication use or withdrawal.

#### *Intervention*

##### 

**Brief Intervention course** A one day course held by two headache specialists (ESK and CL) including lectures about emergency “red flag” headache, migraine and tension-type headache and chronic headaches with focus on MOH were given to the GPs. The content of the BI course was partly based on feedback from a random sample of 30 clinically active GPs at the Department of General Practice at the University of Oslo, regarding which headache-related problems they would like more information on. A two-hour presentation of the BI scheme with practical instructions exemplified by role-play was given. The estimated time for an average BI scheme was expected to be 9–10 minutes. All six GPs participated in a role-play of the BI after an initial demonstration by the two headache specialists. The group then discussed the BI scheme.

##### 

**Figure 2:**
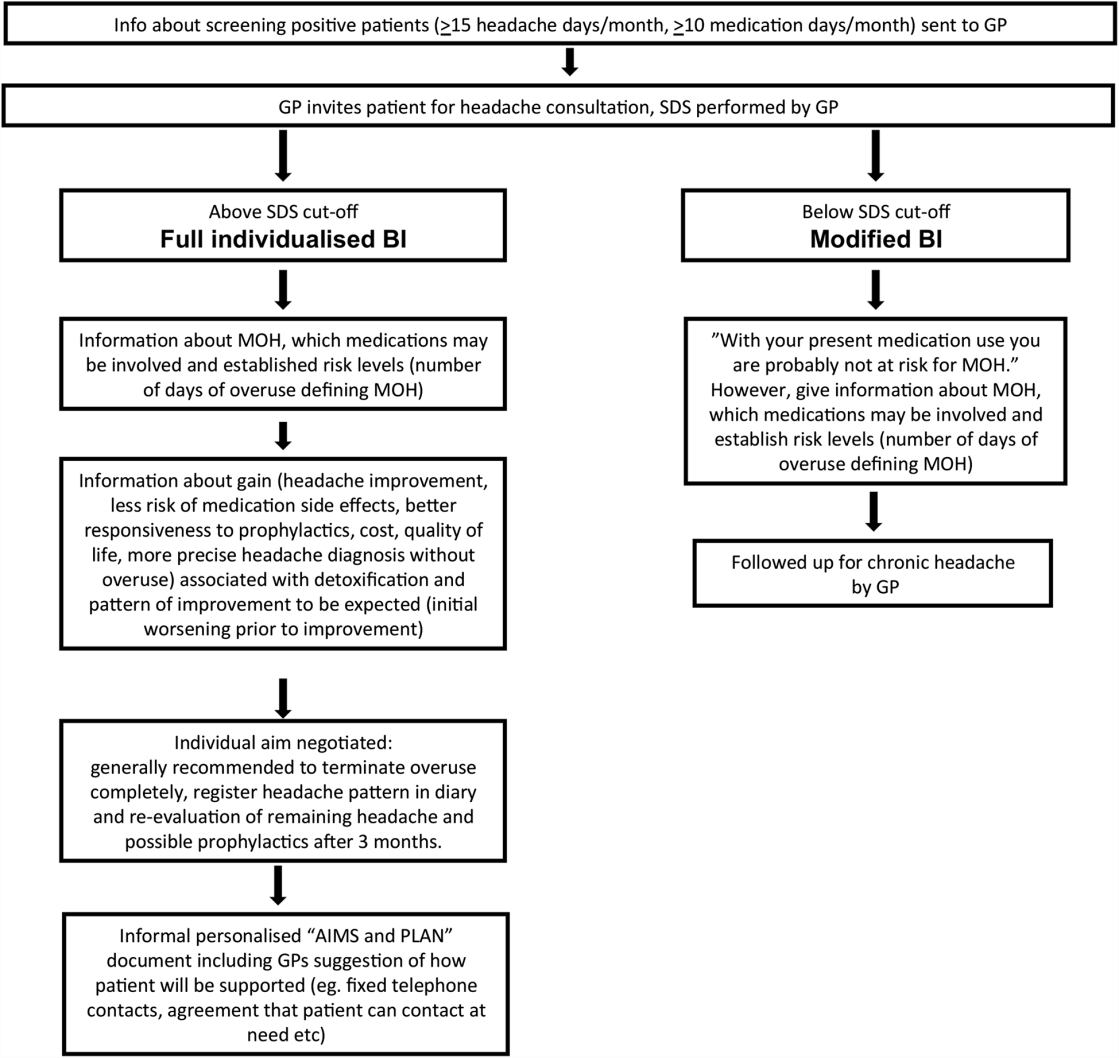
Flowchart of GPs brief intervention for MOH strategy.

**Brief Intervention scheme** The BI strategy (Figure 2) taught to the GPs consisted of:

1. The SDS questionnaire (Table 2) to identify patients at risk for MOH, (SDS cut-off values: females: ≥5, males ≥4). Inform patients above the cut-off that he or she was identified as being at risk for MOH based on Norwegian studies from the general population [26,27]. Patients below cut-off values were given a short version of the full BI.

**Table 2:** The five questions of the severity of dependence scale (SDS) adapted for headache medication such that “your drug” in the original scale was substituted for with the relevant headache medication

1.	Do you think your use of your headache medication was out of control?
	(Never/almost never=0, sometimes=1, often=2, always/nearly always=3)
2.	Did the prospect of missing a dose make you anxious or worried?
	(Scoring as for question 1)
3.	Did you worry about your use of your headache medication?
	(Scoring as for question 1)
4.	Did you wish you could stop?
	(Scoring as for question 1)
5.	How difficult did you find it to stop or go without your headache medication?
	(Not difficult=0, quite difficult=1, very difficult=2, impossible=3)

Each item is scored on a 4-point scale (0–3), and the total maximum score is 15.

2. Short structured information about MOH and the association between medication overuse and chronic headache was given using a short standard information folder developed by the study group.

3. Specific individualised information and advice regarding reduction of acute headache medication was discussed and given based on the individual SDS score. The discussion aimed towards achieving a decision by the patient that he/she would cut down the offending medication, an agreement about how the GP could support and a concrete plan.

The BI incorporated clear directive advice, but focus was also on increasing patients’ insight and awareness regarding overuse, using aspects from motivational interviewing techniques. The primary aim of the BI was to introduce behaviour change perspectives and discussions in an empathic and collaborative manner. It was designed to be relatively short and easily linked to the SDS score. Bien et al. pointed out feedback on personal risk and highlighted the following common motivational elements of effective brief interventions; *“Elements of an effective BIs include Feedback on personal risk, emphasis on Responsibility, clear Advice, a Menu of change options, clinician Empathy, and facilitation of patient Self-efficacy.”*[39].

#### *Headache classification, interview and follow-up*

##### 

**Headache classification** The headaches were classified according to explicit diagnostic criteria of the ICHD-II and it‘s relevant revisions [12-15].

##### 

**Headache diary** A validated headache diary [40] was used in order to prospectively record data on headache frequency, headache intensity (VAS) and medication use. The completion of the diary for a two week period was required at baseline and at three months. A written instruction for the completion of the diary was mailed to participants.

##### 

**Three months follow-up** All patients included in the intervention part of the study were invited to a semi-structured headache interview and clinical examination by a GP specifically trained in headache interview techniques (ESK).

#### *Outcomes*

To evaluate;

• Logistics of patient recruitment including extraction of patients from GPs lists

• Practicality and contents of the BI course as experience by the GPs

• The utility of the SDS and the BI procedure within everyday clinical practice

• Responder rate, completion rate of screening questionnaire and follow-up rate

• Acceptability and compliance of a headache diary with only written instructions sent by mail prior to the consultations

• Acceptability for the SDS and BI among patients

• The format of the three months interview

• Other issues that should be considered for a RCT as identified by patients and/or GPs

The evaluation of the screening questionnaires, headache diaries, BI, BI course and three months follow-up from patients and GPs were not collected in a formal qualitative way, but were given as individual written and spoken feedback after the study was ended by mail, e-mail or phone. Both patients and GPs were encouraged to identify problems and give feedback during and after the study. The feedback will only be presented as direct quotes.

Patient-related outcomes were collected as a part of testing the logistics, but were not a main outcome. The primary and secondary outcomes for the full RCT have been predefined in the study protocol and based on international consensus independently of this feasibility study [16,41,42]. The data from this pilot study will not be pooled with data from the RCT.

#### *Sample size*

We found one CME group (six GPs) to be reasonable to get appropriate information and feedback about the GP perspectives of the methodology and logistics of the study. According to the Norwegian Medical Association, the average number of listed patients per GP is approximately 1200. Using a minimum estimate of 1000 patient per GP gives us an estimate of approximately 10 patients with MOH per GP. With an educational guess of 40% responder rate without reminders that would be 24 MOH patients included.

#### *Statistics*

Only descriptive statistics were used (SPSS version 20.0). Registration of electronic data from the semi-structured interviews was done by using Snap Survey (Snap Survey, London, UK).

#### *Ethics and data security*

The study was approved by the Regional Committee for Medical Research Ethics and the Norwegian Social Science Data Services (NSD). All participating patients and GPs gave informed, written consent. All data were kept anonymously.

### Results

**Figure 3:**
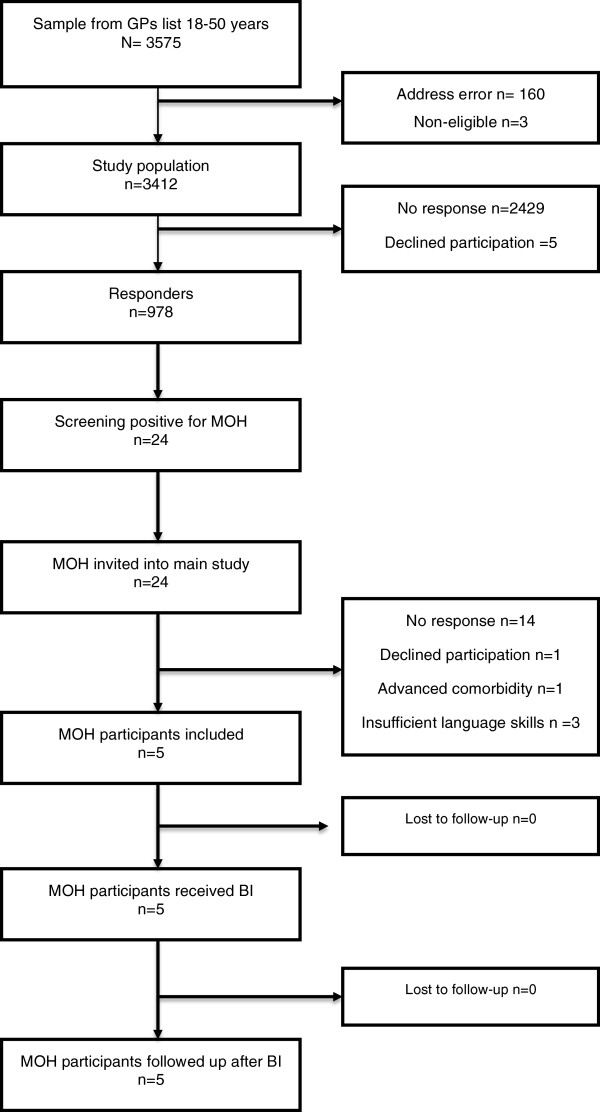
Flowchart of the participating patients.

The participant, intervention and flow details are summarised in the flow diagram depicted in Figure 3. The response rate of the screening questionnaire was 29%.

Twenty-four persons screened positive for chronic headache (≥15 days of headache/month) and 10 ≥ medication days/month and were invited to the intervention part of the study. Of these, five persons filled out the headache diary for two weeks and were included. Five out of six GPs completed a BI for MOH.

All five patients included, received BI, and none were lost to follow-up.

#### *Patients*

The screening questionnaire was complete in over 98% of received questionnaires.

Headache diaries were complete for all five patients at both baseline and the three months follow-up.

The patients reported a high degree of acceptability of the methodology and did not seem provoked by the questions and comments about their medication intake.

Individual comments from patients:

“The screening questionnaire was self-explaining and easy to understand, and the written instructions made the headache diary easy to complete, but I think two weeks fulfilling is the upper limit for completeness when no physicians have told you why the diary is so important!”

“I didn`t feel provoked or stressed when my GP asked me about my medication use in this way, although this is a touchy issue.”

“Individual feedback on medication use is very good; all the information in the media does not concern me as an individual.”

”For a long time I have considered my paracetamol use to be too high but I didn’t know that it could worsen my headache.”

“Nobody has ever told me about the risk of medication overuse headache, the individual information was very clear and relevant for me as a chronic pain patient.”

Table 3 shows patients characteristics at baseline and three months follow-up. The mean SDS score for the five patients included was 6. Of our five patients, four had reduced medication days at follow up and four improved regarding headache days.

**Table 3:** Patient characteristics at baseline and after brief intervention (three months follow-up)

**Patient**	**Gender**	**Age**	**Overused medication at baseline**	**Headache days before BI**	**Headache days before BI (diary)+**	**Headache days after BI**	**Headache days after BI (diary)+**	**Medication days before BI**	**Medication days before BI (diary)+**	**Medication days after BI**	**Medication days after BI (diary)+**	**Headache diagnoses at follow-up**
**1**	Female	45	Paracetamol/Ibuprofen	30	30	30	30	30	30	30*	30	Cervicogenic
**2**	Male	27	Paracetamol/Ibuprofen	25	26	20	18	22	22	5	4	CTTH
**3**	Female	45	Paracetamol	30	30	18	16	30	30	2	2	CTTH
**4**	Female	29	Paracetamol/Ibuprofen	30	30	22	22	30	30	14	14	CTTH + migraine
**5**	Female	35	Combination analgesic (Acetylsalicylic acid/Codeine/Caffeine)	18	18	14	14	18	18	12*	12	ETTH
**Mean**		36		27	27	21	20	26	26	13	12	

BI; brief intervention, CTTH; chronic tension-type headache, ETTH; episodic tension-type headache.

+Headache days and medications days taken from 2 week headache diary.

*Two had completely terminated medication for 2 months but restarted before 3 months follow-up.

#### *General practitioners (GPs)*

None of the physicians had previously received specific training in the handling of MOH. The BI course was held according to the predefined timeframe of eight hours.

The GPs reported the BI as being feasible to implement within a busy practice and that the BI represented a new instrument for communication with these patients*.* During the introduction to the course, participating physicians were anxious about the topic of medication overuse, especially regarding questions on dependency, as possibly being of a very sensitive and thereby provoking nature for the patients. This notion disappeared during the course and this was further strengthened by the feedback after the individual patient intervention.

The time used on the BI scheme was reported between 7 and 12 minutes and well within the normal consultation time.

Individual comments on the BI and the BI course:

“The BI course was informal and practice-relevant.”

”Management of chronic headache patients has always been difficult, but seems easier after this course.”

“The SDS was a great tool to use in the consultation.”

“The SDS and BI strategies made it easy to talk to the patient about her medication use.”

“The SDS was simple to use in practice and only required about 1 minute of consultation time.”

“I had expected some negative feedback from my patient bringing the subject of medication overuse into the consultation, but the SDS and BI really helped us both and made the communication quite easy.”

#### *Three months follow-up*

The time used for the three months follow-up was between 42 and 63 minutes for these five patients. The five patients completed all the additional self-reported questionnaires. The time of completion ranged from 17 minutes to 44 minutes. One of the patients thought there were too many questionnaires that explored the same aspects of self-reported quality of life.

Using direct data entry (Snap Survey, UK) by the interviewer during the interview itself was found to be difficult to adapt to the different ways the patients had of presenting their history. Completion of a structured questionnaire on paper with subsequent data entry after the interview was felt to be more optimal.

#### *Software and data handling*

The developed extracting software (from Mediata Ltd.) used for capturing data from GPs lists performed well and no problems were seen in generating patient lists.

Some minor technical problems with the data entry using Snap Survey were seen and could be corrected.

### Discussion

We have demonstrated that BI for MOH carried out by minimally trained GPs seems feasible and acceptable by both patients and GPs. The main focus was to test methodology and logistics, not patient related headache outcomes.

The responder rate was low (29%), and we included fewer patients than expected. Before the study, we decided that no reminders should be sent to patients because the main aim of this study was to test methodology, acceptability and practicability. We also decided not to include a control arm in the pilot trial, as this not was an efficacy trial. The low number of MOH participants and lack of control group may of course lead to selection bias and low generalization, which suggests the need for increasing individual responder rate and recruitment in a RCT to secure external validity of our findings. The responder rate found here was lower than in a previous study using the same screening questionnaire [6]. The number of reminders used will always be a compromise between cost and gain. In a study focusing on recruiting patients for an intervention, representativity of the sample is important but, providing recruitment is not biased between the study arms in a later study, the percentage responder rate is of less importance than for epidemiological studies. The screening questionnaire was complete in over 98% of received answers, so this seems to have been well understood and accepted. Although the responder rate was low, 24 screening-positive MOH patients were in the range of what we expected. Only 10/24 responded to the invitation into the main study. There was no information in the invitation letter about this being a medication withdrawal study so a possibly negative attitude to this should not have affected the recruitment. We screened all patients on the GPs patient lists instead of relying only on the GPs knowledge of their previous headache patients. Through this screening we expected to reach possible chronic headache patients who might not be known as such by their GP.

The age range of patients (18–50 years of age) was chosen in order to target the highest number of patients with chronic headache, as the prevalence is lower in younger people and older people have a higher frequency of co-morbidities. We chose an upper age limit of 50 years since data from the Norwegian prescription database [43] indicate that there is an increase in the use of various relevant drugs (notably anti-hypertensive and cardiovascular drugs) from 50 years of age onwards.

The headache diary and the written instructions were reported to be acceptable from the patients actually returning it, and recent studies using a similar headache diary and written instructions before first consultation found high usefulness, acceptability and comprehensibility of the diary as well as good compliance and completeness of data [44]. There was clear advice from the patients not to use the headache diary for periods over two weeks since this might reduce compliance. This period may seem too short for infrequent forms of headache, but in our sample of chronic sufferers this should not be a problem.

The evaluation of GPs and patients were not collected in a formal qualitative way, but were given as individual written and spoken feedback after the study was ended. Given the fact that the feedback was quite similar with no major differences regarding the GPs experience of their own patients as being simple or complex, and with no differences between patients managing medication withdrawal or not, we conclude, although few included patients that the BI for MOH seems feasible.

A Danish study has shown that feasibility, acceptability and implementation of screening and brief intervention programs for alcohol overuse in primary care may cause more problems than they solve for some GPs because it might be problematic to incorporate a in a busy daily practice [45]. The feedback from our pilot group of GPs indicated that the SDS and BI were feasible and considered clinically relevant. Our estimated time of 9–10 minutes matched the 7–12 minutes the GPs used on the BI scheme, and all GPs found this easy to do within normal consultation time, even though two of the GPs didn`t know their MOH patients in advance as these patients had never consulted their GP for headache before. 7–12 minutes is well within the normal Norwegian GP consultation time of about 15–20 minutes, in addition we might expect the GPs using less time when more familiar with the BI scheme.

There is still much discussion as to whether MOH represents dependency [20-24]. Being fully aware of this and the potentially problems with stigmatizing these patients, we used the SDS score, not as an attempt to define dependency, but rather as an aid for the GPs to focus on individual risk of medication overuse [26,27]. The GPs did not use the SDS as a universal screening and case finding instrument, it was only used in the BI consultation as an educational tool to give personal feedback. This personal feedback on a risk score is what probably makes the BI scheme more effective than just simple advice [46]. During the BI course, the GPs were anxious about the topic of medication overuse, especially regarding questions on dependency, as possibly being of a very sensitive and thereby provoking nature for the patients. This worry was reduced after the study but suggests the importance of further focus on the experiences of the GPs in the planned RCT with a formal qualitative part.

We had tested the Snap Survey data collection form many times and in many different settings ahead of this study, but still found new errors during the interviews and when reading the data into SPSS. This was not due to the Snap Survey software itself but was a consequence of how the questions and response alternatives were formulated. Such problems may in many cases not have been detected without a pilot trial.

The three months follow-up interviews and additional questionnaires were all performed as planned. The patients reported the questionnaires to be acceptable, one patient though there were too many questions about quality of life and headache-related quality of life [47-49]. However, these instruments have very widespread use and acknowledged validity for assessing quality of life and headache related quality of life and impact and their use is therefore important in order to compare outcomes with other studies.

The patient data support the importance of performing an RCT to assess efficacy of the BI. Two of the patients had completely terminated medication for two months but relapsed before three months follow-up because none of them had received appropriate information from the BI scheme about the importance of staying medication free until the three months follow up.

As a consequence of the nature of this feasibility study we will use previous results from a study from Akershus University Hospital [32] as the basis for the power calculation and sample size required in the RCT. Another reason is that results of pilot studies can potentially mislead sample size or power calculations in full scale RCTs because results may already be biased because of the limited sample size in most pilot studies [35]. None of the data from this study will be used in the RCT to avoid any bias.

In summary, findings suggesting changes in the planned method for the RCT were the need for at least one, and probably two reminders for both the screening questionnaire and the invitation into the intervention part of the study. In addition, since our contacts with the GPs in the pilot study suggested that more than three patients per GP would be difficult to achieve for practical reasons, we must increase the number of GPs included in an RCT. The patients need to receive appropriate information about the importance of staying medication free until the three months follow-up.

We have already published the study protocol with most of these modifications incorporated [42], but nevertheless, we regard the specific methods to be of separate interest, and this feasibility study underlines the importance of doing a proper feasibility and/or pilot study to avoid too many unexpected flaws in a full RCT.

### Conclusion

In this pilot study, BI for MOH seems feasible and acceptable by both patients and GPs. We intend to test the efficacy of BI for MOH in a double-blind pragmatic cluster-RCT since this is a simple intervention with a potential to reach many suffering patients at the lowest effective health care level (GP).

## Competing interests

The authors declare that they have no competing interests.

## Authors’ contributions

CL had the original idea for the study and together with JS, MBR and ESK planned the overall design. ESK prepared the initial draft and was the main author of the present manuscript. ESK and CL carried out the pilot study. MBR supported in the design of the study and with scientific input regarding headache. All authors have read, revised and approved the final manuscript.
